# Supplementary material for: A scalable algorithm for structure identification of complex gene regulatory network from temporal expression data
Source: BMC Bioinformatics. 2017 Jan 31;18:74. doi: 10.1186/s12859-017-1489-z (PMC5294888; doi:10.1186/s12859-017-1489-z)
Supplement: Additional file 1 — Text S1. System requirements for running DMI. (PDF 52.0 kb) [file 12859_2017_1489_MOESM1_ESM.pdf]

System requirements for running DMI

Currently the code has been tested using 64-bit MATLAB 2016a on a Mac machine with 16GB memory. If you encounter any memory error, our suggestion is to 1) increase the system memory to 16GB; 2) switch to a 64-bit operating system as well as a 64-bit version of MATLAB.
