# Supplementary material for: A scalable algorithm for structure identification of complex gene regulatory network from temporal expression data
Source: BMC Bioinformatics. 2017 Jan 31;18:74. doi: 10.1186/s12859-017-1489-z (PMC5294888; doi:10.1186/s12859-017-1489-z)
Supplement: Additional file 2 — Text S2. Parameter tuning guideline for DMI. (PDF 64.4 kb) [file 12859_2017_1489_MOESM2_ESM.pdf]

Parameter Tuning Guideline for DMI

Here we provide the guideline for parameter tuning of DMI. First, to impose a constraint on the number of parent nodes for node i, $\xi_i (i = 1,...,n)$ are introduced into our algorithm formulation. One does not need to manually specify the value of each ξ_i. We suggest to set ξ_i as the product of a constant coefficient $c \in (0,1)$ and the indegree of node i in the background network. Our computing code will automatically calculate the indegree of each node in the background network so users only need to modify c if he/she wants to allow less or more incoming edges to node i. In all our simulation studies and real data application, $c = 0.7$ was used and turned out to be sufficiently large due to the sparsity property of complex networks. Second, α and β are the penalty coefficients for hub structures (A) and sporadic sparsity structure (B), respectively. These two parameters are the major tuning parameters that have to be determined using cross-validation, which is a common practice for sparsity penalization. For real data analysis in this study, $\alpha = 0.08$ and $\beta = 0.22$ were used. Third, parameter ρ plays a role in controlling the step size of each iteration to guarantee the monotonic decreasing. However, it is possible to automatically adjust ρ in the DMI algorithm implementation so users can simply supply a positive number (e.g., $\rho = 10$) and it will work.
