# Supplementary material for: A scalable algorithm for structure identification of complex gene regulatory network from temporal expression data
Source: BMC Bioinformatics. 2017 Jan 31;18:74. doi: 10.1186/s12859-017-1489-z (PMC5294888; doi:10.1186/s12859-017-1489-z)
Supplement: Additional file 3 — Table S1. Computational settings for the competing methods under comparison. (PDF 56.5 kb) [file 12859_2017_1489_MOESM3_ESM.pdf]

Table S1: Computational settings for the competing methods under comparison.

Methods	Settings
GENIE3	default setting in its MATLAB package
Jump3	noiseVar.sysNoise=0, noiseVar.obsNoise=0.01
CLR	method='stouffer', n = 10, k = 3
SITPR	alpha=0.05
ARACNE	default setting in its R package
TimeDelay-ARACNE	delta=1, likehood=1.2, norm=2, logarithm=0, thresh=0, ksd=0, tolerance=0.13, adj=TRUE
PCC	default setting for MATLAB function ‘corr’
MINET	default setting in its R package for the Maximum Relevance Minimum Redundancy method
TIGRESS	R=500, alpha=0.1, L=2
