# Supplementary material for: A scalable algorithm for structure identification of complex gene regulatory network from temporal expression data
Source: BMC Bioinformatics. 2017 Jan 31;18:74. doi: 10.1186/s12859-017-1489-z (PMC5294888; doi:10.1186/s12859-017-1489-z)
Supplement: Additional file 4 — Table S2. Additional experiment results of algorithm performance evaluation of DMI on a real background network with 2768 nodes. We retrieved the UCSC background network for human from the RegNetwork database (http://www.regnetworkweb.org/), and then selected the highly confident interactions as the activated edges (RegNetwork provides the tool to select edges of different levels of confidence). The number of such activated edges is about 1/6 of the total number of edges. In this way, we avoid introducing or removing any random edge into the background network. (PDF 59.7 kb) [file 12859_2017_1489_MOESM4_ESM.pdf]

Table S2: Additional experiment results of algorithm performance evaluation of DMI on a real background network with 2768 nodes. We retrieved the UCSC background network for human from the RegNetwork database (http://www.regnetworkweb.org/), and then selected the highly confident interactions as the activated edges (RegNetwork provides the tool to select edges of different levels of confidence). The number of such activated edges is about 1/6 of the total number of edges. In this way, we avoid introducing or removing any random edge into the background network.

Method	Size	SN	SP	ACC	F-measure	MCC	AUC
DMI	2768	0.67±0.01	0.96±0.00	0.93±0.00	0.79±0.01	0.63±0.01	0.82±0.01
