# Supplementary material for: A scalable algorithm for structure identification of complex gene regulatory network from temporal expression data
Source: BMC Bioinformatics. 2017 Jan 31;18:74. doi: 10.1186/s12859-017-1489-z (PMC5294888; doi:10.1186/s12859-017-1489-z)
Supplement: Additional file 5 — Figure S1. In-degree distribution of the A549 GRN. The power-law model fitting result is labelled in red. (PDF 65.6 kb) [file 12859_2017_1489_MOESM5_ESM.pdf]

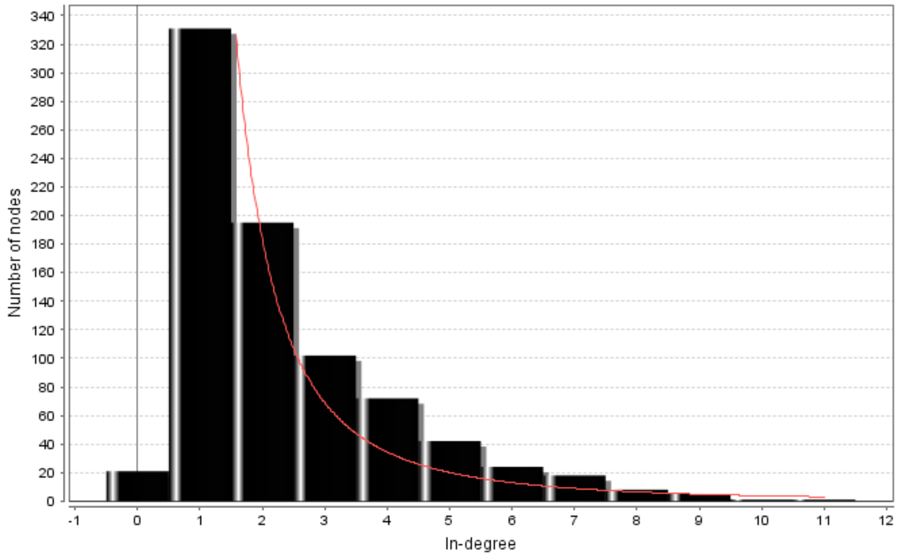

Figure S1. In-degree distribution of the A549 GRN. The power-law model fitting result is labelled in red.
