# Supplementary material for: A scalable algorithm for structure identification of complex gene regulatory network from temporal expression data
Source: BMC Bioinformatics. 2017 Jan 31;18:74. doi: 10.1186/s12859-017-1489-z (PMC5294888; doi:10.1186/s12859-017-1489-z)
Supplement: Additional file 6 — Table S3. Basic network statistics of the A549 GRN. (PDF 138 kb) [file 12859_2017_1489_MOESM6_ESM.pdf]

Network analysis of the GRN in A549 human respiratory epithelial cells during influenza H1N1 infection

The GRN structure identified using the proposed DMI algorithm is a typical complex network, as suggested by the network analysis results listed in Table S1 and Fig. S1. In particular, the clustering coefficient of this network is 0.032, which is significantly higher than that of random networks (~1.5×10^{-5}) [1]. Also, the characteristic path length of the A549 GRN is 4.840, which is comparatively small and may suggest an efficient propagation of regulatory signals in a directed manner. Moreover, the node degrees of the A549 GRN follows the power-law distributions as shown in Fig. S1. Let y denote the number of nodes and x denote the node degree, the power law model is given as $y = ax^{-b}$. By fitting this model to the A549 GRN data, we obtain $\hat{b} = 2.413$ (with $R^2 = 0.837$ computed on log-transformed values). Since $2 \leq \hat{b} \leq 3$, we know that A549 GRN is scale-free [1, 2].

Table S3. Basic network statistics of the A549 GRN.

Clustering coefficient	0.032
Network diameter	11
Network radius	1
Shortest paths	36,664
Characteristic path length	4.840
Avg. number of neighbors	4.371
Multi-edge node pairs	89

References

1. Barabasi, A.L. and R. Albert, *Emergence of scaling in random networks.* Science, 1999. **286**(5439): p. 509-12.
2. Newman, M.E.J., *The structure and function of complex networks.* SIAM Review, 2003. **45**(2): p. 167-256.
