# Supplementary material for: A scalable algorithm for structure identification of complex gene regulatory network from temporal expression data
Source: BMC Bioinformatics. 2017 Jan 31;18:74. doi: 10.1186/s12859-017-1489-z (PMC5294888; doi:10.1186/s12859-017-1489-z)
Supplement: Additional file 7 — Figure S2. Subnetwork structure centered at ‘CEBPB’. The subnetwork structure is identified using the DMI algorithm based on the time-course expression data from human A549 cells in response to IAV infection. Blue arrows stand for negative regulatory relationships, and yellow arrows stand for positive regulatory relationships. (PDF 130 kb) [file 12859_2017_1489_MOESM7_ESM.pdf]

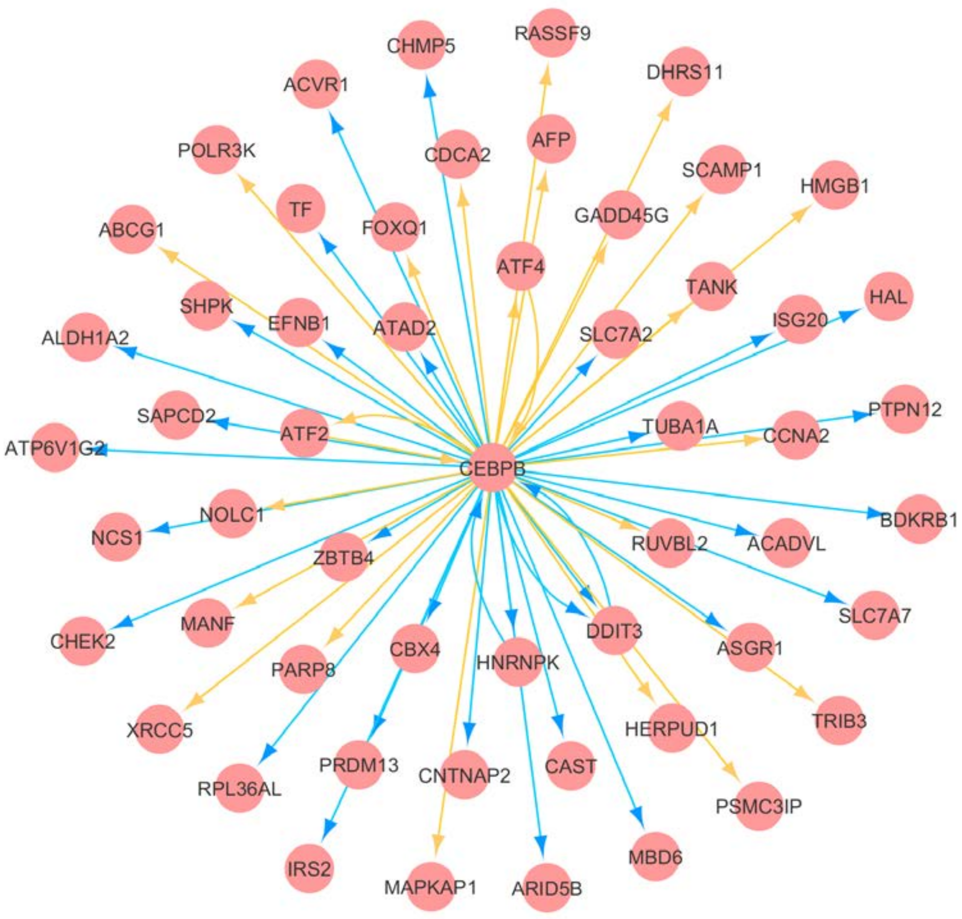

Figure S2. Subnetwork structure centered at CEBPB. The subnetwork structure is identified using the DMI algorithm based on the time-course expression data from human A549 cells in response to IAV infection. Blue arrows stand for negative regulatory relationships, and yellow arrows stand for positive regulatory relationships.
